# The Hospital World

**Published:** 1911-06-03

**Authors:** 


### THE HOSPITAL WORLD.

### The late Sir Wilfred Hepton.

The sudden death by drowning, at Argelès, in the Pyrenees, of Sir Wilfred Hepton came as a shock to everyone in Leeds. He was a prominent man in the city, having been formerly Lord Mayor, and was known in institutional circles as Chairman of the Leeds Public Dispensary. He also figured in the recent discussion as to the limitation of the number of appointments held by the members of the honorary medical staff of the Leeds Infirmary. For some reason or another he strenuously opposed this alteration in the rules. He was known in Leeds University circles, and gained his knighthood in 1907.

### The Foundress of the Queen's Hospital for Children.

Mr. J. Miller, last week at the annual meeting of this hospital, excited much interest by saying that he would like to refer to a name that had been included that day among the vice-presidents, that of Miss Phillips, the foundress of the hospital. The life devotion of that lady to the charity was beyond commendation, and it was a satisfaction to everyone connected with the hospital that her name had been included among the list of vice-presidents. He was sure, too, that it was a matter of great satisfaction to them to know that a lady whom they all esteemed so highly had successfully passed through a very serious illness. He added that a special acknowledgment was due to Mr. Glenton Kerr, another worker, for the valuable work he did as secretary.

### The Late Treasurer of the Royal Hospital for Incurables

By the death of Mr. Herbert John Allcroft, the Royal Hospital for Incurables, Putney Heath, has suffered an almost irreparable loss. For many years Mr. Allcroft (following the example of his father, Mr. John Derby Allcroft) served as treasurer and chairman. Mr. Allcroft threw himself most devotedly into many works of philanthropy, and no charity had more of his time and energies than had the Home for Incurables on Putney Hill. At the time of his death Mr. Allcroft was in his forty-sixth year. He passed away after a long and most painful illness, borne with great patience and fortitude. Only so recently as May 4 a long letter, written by his own hand, was received by the Secretary of the Royal Hospital for Incurables, in which was enclosed a cheque for £141 15*s*., being a donation of 100 guineas from himself, 25 guineas from his wife, and five guineas from each of his two children, towards the Festival Dinner Fund. It was a melancholy coincidence that Mr. Allcroft should breathe his last on the day when an election of candidates for the Royal Hospital for Incurables was taking place, for it was his custom to take the chair at these half-yearly elections. The last election (the date of Mr. Allcroft's death) was May 26, and the Deputy Chairman, Mr. Thomas W. Wickham, in opening the proceedings, referred very sympathetically to the critical illness of Mr. Allcroft and to his enforced absence; at the close of the proceedings, two or three hours later, it was Mr. Wickham's sad duty to announce that during the last hour he had received a telegram announcing Mr. Allcroft's decease. As the *Times* recalled, Mr. Allcroft was born on July 10, 1865, and was educated at Harrow. He read for the Bar in the chambers of Mr. (now Lord Justice) Vaughan Williams, and in 1890 was called by the Inner Temple. In 1893, on the death of his father, who was largely interested in the firm of Dent, Allcroft and Co., he inherited the extensive estate near Craven Arms, which his father had acquired by private purchase, and which formerly belonged to the Earls of Craven. He was a diligent landlord, spending most of each year on the property, and for many years hunted with the Ludlow Foxhounds. He served the office of High Sheriff in 1893, and was on the Commission of the Peace for the county. He married on December 15, 1900, Margaret, only daughter of the late General Sir William Russell, C.B., and leaves a son and daughter. The former, John Russell Allcroft, who inherits the property, was six years old last week.

We understand that the Royal Hospital for Incurables was represented at the funeral of its late treasurer by Mr. Thomas W. Wickham, the Deputy Chairman, Mr. Charles Cutting, the Secretary, and the Rev. Digby Critten, the Chaplain.

### Hospital Flags and the Right to Fly Them.

On May 26, the Queen's birthday, the flag presented to the Royal Dental Hospital by the Students' Club was hoisted for the first time by Mr. John Hampton Hale, Chairman of the Committee of Management. The flagstaff and Union Jack have been subscribed for by the students of the Medical School attached to the hospital. Mr. Hale said that the Queen's birthday was a very appropriate occasion for the first hoisting of the flag, it being the first year that the hospital has enjoyed her Majesty's patronage. The Coronation, no doubt, will raise again the technical question as to what flag or flags a hospital is strictly entitled to fly. Questions on the subject have been addressed to us recently, and we hope to give the officially correct answer next week. It has been a matter of research to find and sometimes to reconcile the heraldic and legal technicalities of this question. It is a far nicer point than is generally supposed.

